# The Use of Urban Parks by Older Adults in the Context of Perceived Security

**DOI:** 10.3390/ijerph19074184

**Published:** 2022-03-31

**Authors:** Kinga Kimic, Paulina Polko

**Affiliations:** 1 Department of Landscape Architecture, Institute of Environmental Engineering, Warsaw University of Life Sciences—SGGW, Nowoursynowska Street 159, 02-776 Warsaw, Poland; kinga_kimic@sggw.edu.pl; 2 Security Studies Department, Faculty of Applied Sciences, WSB University, Cieplaka Street 1c, 41-300 Dabrowa Gornicza, Poland

**Keywords:** urban greenery, older adults, perceived security, fear factors, age-friendly parks, active-friendly parks, physical activity, inclusiveness, Poland

## Abstract

The perception of urban greenery is determined by many aspects, including the personal security of different groups of city dwellers. The objective of this study was to investigate if there are differences between the sense of security of older adults and other groups of urban park users, and which factors play an important role in the evaluation of personal security and thus determine the use (or not) of parks. A survey questionnaire was administrated to a sample of randomly selected park users in Poland (*n* = 394), including seniors (*s* = 69). The results show statistically significant differences in security perception between respondents under the age of 60 and those over the age of 60 in the case of all questioned factors. At the same time, all of them are important for a sense of security in older adults. This knowledge is crucial for designing more inclusive and age-friendly urban parks, which should meet the needs and expectations of older adults and encourage them to engage in more activity.

## 1. Introduction

Urban greenery provides a wide range of social benefits for city dwellers [1]. These benefits, which may improve people’s quality of life, become of key importance and should be taken into account in the planning and design process of urban parks functioning as open public spaces free and available to everyone. This approach is crucial nowadays, especially for older adults in the context of increasing their health and well-being. Demographic aging is a global trend resulting from a sustained change in the age structure of the global population, driven by rising levels of life expectancy. The share of 60+ aged people in the total population is growing rapidly, especially in the 21st century. At the same time, the number of threats, such as reduced physical and mental performance, the occurrence of diseases and injuries, etc., is increasing with age, which translates directly into increasing motor impairment and progressive disability in aging [2]. It is estimated that the share of people that are aged 65 or more, recognized at 727 million people in 2020 (9.3% of the population), will more than double over the next three decades, reaching over 1.5 billion in 2050 (16.0% of the population). It is also expected that in the coming decades, a large percentage of the increase in older adults will occur in urban areas [3]. The above-mentioned tendencies are observed also in Poland; a strong increase in the share of the elderly in the population has also been observed: in 2005 it was 17.2%, in 2006 it increased to 17.2%, and in 2019 it was as high as 25.3%. The forecasts for the coming years include further growth: in 2030, it will reach 28.0%, in 2040, about 32.0%, and in 2050, over 35.0%. In 2050, people of senior age living in cities are expected to constitute 23.5% of the Polish population. The aging causes changes not only in the economic sphere, but also to lifestyles, forming new cultural patterns, especially in terms of how people spend their free time. According to data from the Central Statistical Office, 55.1% of Polish older adults go for a walk or spend time outdoors (including urban parks) at least once a week [4].

### 1.1. Aging-Friendly and Safe Urban Greenery

The creation of conditions for active and friendly aging of the population [5], including aging-in-place [6], should be focused on using the potential of urban green areas for enhancement of both the mental and physical health of older adults. It is in line with approaches promoted by the WHO relating to activity by optimizing opportunities for health, participation, and security in order to enhance quality of life and well-being [7]. Healthy aging aims to keep the elderly in good condition and independence for as long as possible [8,9,10,11]. The creation of places where older adults can be socially and physically active is a global health priority [12]. Green areas, mentioned as age-friendly features [7], play an especially important role in this context. Green areas located close to the place of residence become positively perceived neighborhood parks and more frequently visited by 60+ aged people [13]. With respect to “healthy aging” and especially “active aging” [14], urban parks have been identified as important places of physical activity for senior city residents [15]. They are places of walking, rest and recreation, and sport [16,17,18,19,20], and provide an opportunity to be active daily [13,17,21]. They also ensure that older adults have contact with nature [22,23,24] in a highly urbanized space and encourage them to spend more time outdoors [25]. Urban parks may also promote the development of many forms of social interactions [1,26] by establishing bonds with others and participation in social life [20,23,27,28,29,30,31,32,33,34], which is important for maintaining mental health [24,25,30,35,36,37,38,39,40,41,42,43]. Due to those benefits, urban green spaces become free of charge and inclusive spaces for all “public health resources” in cities. However, only safe urban parks may perform all above-mentioned functions at a high level [44]. At the same time, there is a relation between perceived security and physical activity. Older adults especially have different perceptions, needs, as well as desires related to the use of urban green areas than other groups of users [26]. They generally need to feel safe in urban parks in order to rest or spend time actively. In that context, many factors related to security which affect their choice of park, undertaken activities, as well as the duration of their visits [45] should be taken into account in the research on the use of urban parks by older adults.

Security is one of the most important categories that allow for the description of the context of life and the way a person functions [46,47,48,49,50,51]. It is defined as an objective state of non-threat that is subjectively felt by individuals or groups, or the process of reaching that state. This definition includes two perspectives of understanding security: the objective perspective, which is related to external factors essential for a proper life; and the subjective perspective, which is related to an individual assessment of the state of possession or availability of essential goods. In psychology, security is understood as a need, value, belief, and feeling [46,47,49,52,53]. The feeling of security is so significant that it becomes a motivating factor either as a need [46,48] or as a value [49,53]. The problem of security does not exist apart from an individual’s perception and personal experience [47]. Although it is subjective, it plays an important role in assessing the situation and making decisions [48,54]. It can, therefore, be concluded that security in urban green spaces is, and will continue to be, an important social issue. Many studies confirm that a sense of security is recognized today as one of the most important aspects influencing the perceived quality and attractiveness of urban parks [41,55,56]. Otherwise, negative effects such as fear, anti-social behavior, and stress can occur [57]. At the same time, the age of users is important for the way parks are designed and then used [58,59], especially if the main groups of visitors to green areas in cities are older adults [60]. For those groups of adults, it is not only accessibility or proximity, but above all, safety that determines the use of urban parks [24,26,31,58,61].

### 1.2. Security Providing Factors in Urban Parks in Context of Older Adults Activity

Age is often associated a lower level of personal safety and avoiding places in the open air, which is especially observed in 60+ aged people [62], and thus may decrease their physical activity. According to Wiles et al. [63], the sense of security is important for older adults and applies to the park environment. The literature referring to seniors’ assessment of safety in urban greenery and the factors shaping it, including their impact on physical activity, does not give an unambiguous answer to the question of whether they differ in this respect from other age groups. Some authors claim that 60+ aged park users are generally more afraid of dangers in urban green areas than younger ones [26,64,65,66,67,68,69]. Fear generally limits the use of the park for older adults more than that it does for young people [65]. They are also more likely to express concerns about safety and crime [70]. However, the relationship between safety and urban parks is complex and involves many aspects. Individual factors can be critical for how safety problems are approached in the physical environment [71], and the use of this knowledge is important for the planning and design of urban parks.

#### 1.2.1. Visibility

Many aspects of visibility can influence the sense of security in urban parks [72]. Lighting is one of the frequently mentioned factors, usually having a positive impact on the perception of security in public spaces by all user groups and is used as a solution for crime prevention [73,74,75,76,77,78]. Good visibility allows people to observe the surroundings and see potential threats from a distance [73,79,80,81,82]. The time of day, related to the amount of light, has an impact on the perceived safety [83,84,85,86], i.e., strongly shaded areas are usually assessed as dangerous and should therefore be avoided [82,83,84]. The sense of danger increases at night [74,81,87,88,89,90,91,92], which especially affects older adults who are more afraid of using parks after dark so as not to become victims of crime [86]. For this reason, the amount of artificial lighting also becomes an important predictor of the perceived safety in urban green areas [93,94,95,96,97].

Some research confirms that older adults derive considerable pleasure from viewing and being in nature which, in turn, has a positive effect on their well-being and quality of life [98]. However, many park users avoid areas with poor lighting resulting from high a density of trees and understory vegetation [64]. Many studies confirm that the character of vegetation itself can be an important factor affecting perceived personal safety [92,99,100,101,102,103]. Dense and neglected greenery may be assessed as elements which block the escape route in times of danger, create potential hiding places for criminals and undesirable park users [98,104,105,106] and thus contributes to an increase in crime [81,101,107,108,109] and a lower sense of security, especially in older adults [106]. City dwellers prefer parks without dense vegetation and feel safer in areas with high visibility [110]. The anxiety related to the presence of vegetation in parks has been described in many publications [64,104,109]. In connection with the fear of crime, in the case of 60+ aged people, it may limit or even prevent their physical activity [111,112].

#### 1.2.2. Maintenance and Cleanliness of Parks and Park Facilities

Good maintenance of urban parks is crucial to the perception of urban greenery as low-risk areas and allows people of advanced age to feel safer [113,114]. It also determines a positive image of green areas. People tend to avoid areas containing equipment that is damaged [30] because it suggests that park is run down and possibly unsafe [115]. Older adults prefer well-maintained infrastructure and facilities [24] and pay particular attention to the technical condition of facilities such as benches, which are the basic elements ensuring rest [116,117]. However, it is equally important to maintain other furniture, structures, and devices [118]. Well-maintained paths are highly desired by all users of urban parks [26,119,120], especially by older adults [24,106,121,122,123,124,125] for whom poor paving can significantly limit activity in open public spaces [126]. For those with an active lifestyle, the quality of equipment [26], including sports and fitness facilities is also important [127,128]. Additionally, well-kept greenery strengthens the sense of security [62,64,108,118,129] at the same time as increasing the attractiveness of urban parks [57,80,108], which is also desired by older adults [106]. Neglected plants with damaged and falling branches threaten the safety of park users as they can cause injuries or even the death of people who are accidentally hit [130,131,132,133]. Maintaining green areas and other park facilities at a high level, which improves safety and accessibility, is mentioned as one of the most important aspects in designing inclusive urban parks [116] and a form of support in preventing criminal behavior [134].

Many physical factors related to cleanliness can increase the feeling of insecurity in urban green spaces [92,135,136], i.e., disordered physical environment such as dirty or neglected areas, the presence of litter on the ground, overfilled refuse bins, graffiti, damaged equipment, dilapidated buildings, the presence of dog droppings [30,137], as well as vandalism and physical signs of incivilities [97]. These signs of insufficient management of green areas or an excessive number of visitors [138,139] may also strongly impact the perception of security in urban parks [97]. Dirty spaces encourage anti-social behavior and crime [140,141]. The presence of graffiti on park facilities and buildings can contribute to the perception of a lack of safety [80,142]. Some studies also prove that litter on the ground and vandalism may heighten the sense of fear in public spaces [143]. A low level of cleanliness in urban parks is often identified as the reason minimizing the number of visits, regardless of the type of users [144,145], and a factor decreasing their use in physical activity [139]. A clean space without rubbish is important for various groups of park users [24,121,146], especially for older adults [26,106].

#### 1.2.3. Mobility Facilities

Many 60+ aged users of urban green areas pay attention to factors such as the accessibility of the facility and the quality of its infrastructure, especially when related to mobility facilities. Several studies reflect the general assumption that older adults tend to reach parks on foot and walk in the open air mainly for the sake of their health [147,148,149]. Walking, as a moderate-intensity exercise, is the most common behavior in urban parks and can be easily integrated with other activities; therefore, walking is more attractive to older adults than high-intensity exercise [150,151,152]. Since for those groups of park users the sense of security both in relation to their own body and the environment seems to play a fundamental role [153,154], the technical condition of equipment may influence decisions regarding the use of urban green areas. The presence and quality of park paths are important for 60+ aged users [24,106,121], and because they are the basic elements that ensure navigating the terrain, they also play an important role for the safety of their use. The lack of paths, uneven terrain, and poor-quality surface, are seen as problems for many park users [119]. Well-paved and safe paths are highly demanded by older adults [122,123]. A smooth surface and barrier-free pavements can increase their independence and social participation as many of people over the age of 60 have mobility problems [155] and are the most at risk, such as those who are prone to falls [156], or with basic weakness of functions related to movement and balance [157]. At the same time, security may be associated with a sense of frailty and vulnerability [111,158]. Older adults with reduced mobility and/or a history of falls appreciate the smoothness of the pavement in combination with continuous curbs and crossings on pedestrian routes [12]. The lack of amenities in this context may limit their physical activity [159], including the intensity of using parks. Additionally, a general diversity of park topography difficult for 60+ aged users to overcome may be one of specific attributes affecting their use of urban green spaces [160,161] and may have an impact on the perception of the area as safe or not [151]. The availability and safe use of parks are also determined by other features, such as walk slope and presence of mobility facilities, which are particularly demanded by the disabled and older adults [151]. Especially, functional aids also influence their activity. In urban spaces, including green areas, there may be many physical barriers limiting both walks and recreation [162,163]. The lack of basic amenities, such as benches [164], paths, and mobility aids [165], dissuades older adults from being physically active in parks [152]. The lack of access to other amenities, such as ramps (including those with handrails on both sides), especially in the case of 60+ aged users with limited mobility and other disabilities, additionally causes worry about physical safety and discourages visiting green areas [166]. Possible difficulties in moving around the park may be thus the key factors affecting the sense of security [163,167].

#### 1.2.4. External Protection

The security measures applied in urban parks relate to various elements, the most common of which is the use of a fence that acts as a strong psychological deterrent and spatial control element, as well as an element that provides a certain degree of privacy [168,169]. Enclosure, both visual and physical, does not only affect safety, but can also create a calm atmosphere, trigger restorative experiences, and affect the possibility for restoration [170]. The presence of law enforcement agencies, such as the police and guards, video surveillance (closed-circuit television cameras (CCTV)) or compulsory signs has an impact on the safety and reduction of crime in public spaces and green areas. CCTV and improved street lighting are the most well-developed surveillance measures used in urban public spaces [75,171] and are an alternative to the presence of police officers who are not be able to patrol the park at all times [45]. However, parks are considered difficult to monitor because their large size and vegetation limit the view into the open space. Although closed-circuit television often does not cover the entire area [115,172], its use has an impact on reducing the fear of crime [173,174]. Therefore, it is crucial to use various forms of external protection and adapt them to the characteristics of the site. The implementation of the Crime Prevention Through Environmental Design (CPTED) strategy, the aim of which is to reduce the sense of anonymity and strengthen the natural disposition of people to observe the environment, may also be helpful [175,176,177].

### 1.3. Aim of the Study

Based on the literature review, older adults represent a group of park users with special preferences related to the security. However, there is not much information regarding how they differ in relation to other age groups of people visiting urban green spaces. According to our best knowledge, the research in that field has not been conducted and published in the context specific to Poland. Only a few papers concern the sense of security of urban park users and are related to selected aspects, e.g., impact of vegetation [101,167] or CPTED [178]. There is also no detailed research identifying the factors that affect the sense of security of older adults, especially in the context of comparisons with other groups. Taking into account the special needs of older adults, the research on perceived security related to the physical attributes of parks which may affect physical activity of this group of users requires broadening. This applies to aspects such as topography and the presence of paths and equipment which facilitate walkability and ensure the access to urban parks. Another group consists of factors related to the maintenance and cleanliness of urban greenery. Important aspects are also related to visibility as well as external protection.

Therefore, the objective of this study was to investigate which factors were important for shaping security perception (mobility facilities, maintenance and cleanliness, visibility, and external protection), indicated after literature review, play an important role in evaluation of personal security of their users. It was also crucial to highlight how senior age differentiates the perception of security. This paper is the continuation of our previous study [179], which analyzed how gender differentiates the perception of safety in urban parks and showed statistically significant differences in selected factors. Due to the previously indicated gaps in Polish studies, we decided to examine these factors in relation to older adults as a group of park users with special requirements, and are, at the same time, more exposed to threats and thus to social exclusion. The data collected in this study may support designing new urban parks, but also contribute to the modernization of existing urban parks, making them more inclusive. Therefore, we want to confirm the following hypotheses:

**Hypothesis** **1.** *There are differences between the sense of security of older adults and other groups of urban park users.*

**Hypothesis** **2.** *Most factors related to aspects of the presence of mobility facilities, maintenance and cleanliness, visibility, and external protection are more important for older adults’ sense of security as a group of park users with special requirements.*

## 2. Materials and Methods

### 2.1. Participants

This study was conducted on a group of 394 randomly selected adult park users, including 69 respondents over 60 years old (F = 40, M = 29). The sample differed in terms of gender, education, place of residence, access to urban parks and frequency of their use. All sample characteristics details are presented in Table 1.

Most of the respondents (275, 69.8%) declared that they live in cities with public parks. The largest group of respondents, 82.32% of all users and 89.9% of older adults, had access to more than one park. A total of 11.4% of all users and 8.7% of older adults had access to at least one park near the place of living. Only 6.31% of all respondents and 1.4% of older adults declared that didn’t have any parks in their town/city; however, they visited parks in other places. Therefore, the vast majority of respondents, including people over 60 years of age, had no problem with access to urban parks.

Most of the respondents (109, 27.7%) visit parks once a week. In case of the oldest park users, those who visit them once a week were the biggest group; approximately one third of older park users declared this frequency of use (25, 36.2%). However, in the case of the remaining groups of respondents, the situation was different. In the entire research sample, the second largest group of respondents are those who do it sporadically, several times per year (98, 24.9%) and in the case of people over 60, it was the group visiting the park once a month (19, 27.5%). The respondents visiting parks once a month (82, 20.71%) and 2–3 times per week (79, 19.95%) take the next positions among all respondents. In the case of older adults, 20.3% (14) of them declared that they visit urban parks a couple of times per year and 13% (9) of them declared that they visit urban parks 2–3 times per week. The results indicate that seniors used urban parks more often than their younger counterparts.

### 2.2. Selection of Factors Affecting Perception of Security

The implementation of the research aims required, first of all, the identification of factors shaping safety in urban parks based on literature review, as those emphasized as being associated with perceived security related to the use of urban parks and their physical attributes [57,64,65,73,74,89,90,92,111,179]. They were classified into 4 categories according to the scheme presented in Table 2: (1) the presence of mobility facilities, including 3 factors (related to walkability in the park); (2) maintenance and cleanliness, including 6 factors (related to various elements of the park condition); (3) visibility, including 5 factors (determined by the level of illumination and density of greenery); and (4) external protection (related to presence of police patrol or video surveillance and fence).

### 2.3. Questionnaire Characteristics and Procedure

The questionnaire as a research instrument has been used to collect information from the respondents. Its structure, except main demographic data (presented in Table 1), consisted of questions following the above-mentioned categories and their factors selected for the study. The questionnaire asked the participants to rate each of the factors in terms of perceived security on a 5-point Likert scale [180], where 1 meant a very low, and 5 meant a very high impact of the factor. The questionnaire was pilot tested prior to data collection to ensure clarity of the questions and improved.

The quantitative data used for analysis was collected through the survey questionnaire conducted online in the period from March to August 2020. It was aimed at an anonymous group of respondents over 18 years old, randomly selected from users of urban parks. Then, the PS Imago Pro 6.0 program was used to analyze the collected data. A Student’s *t*-test was used to show the significance of differences in responses between under 60 and 60+ aged respondents (*p* < 0.05). The reliability of the ratings of factors was tested with the Cronbach reliability test and yielded a relatively high Cronbach’s alpha = 0.816.

The raw data retrieved from questionnaires were used for statistical analysis purposes in terms of the statistical significance of the differences between the responses of older adults and other age groups of survey participants. The aim was to check whether the group of 60+ aged park users, identified in the research to be particularly vulnerable to exclusion, has a different perception of the importance of selected factors in the context of safety in urban parks. In line with the assumptions contained in the hypotheses, it was first checked which of the indicated factors are the most important for older adults in the context of their perceived security. Then, it was determined how many and which factors had statistically significant differences in their perception of importance for shaping the sense of security in various age groups, in particular, between older adults and younger users of urban parks.

## 3. Results

The respondents’ opinions about the factors influencing their safety in urban parks were important to answer the question of their general sense of safety in these places.

**Figure 1:**
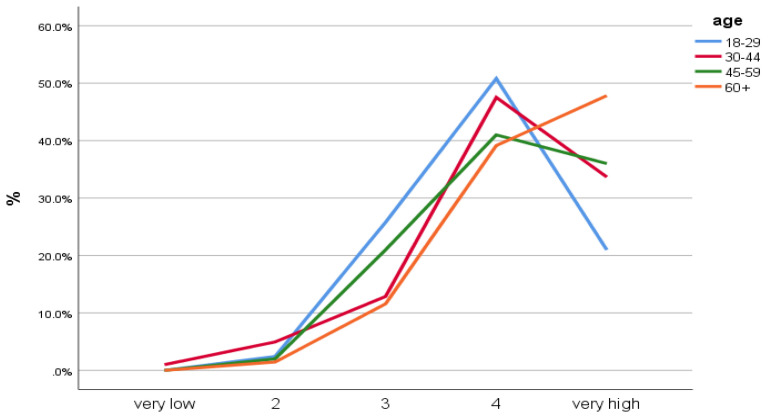
General assessment of the level of safety in urban parks by users (age groups) (elaborated by authors).

When asked about the general assessment of the level of safety in parks on the 5-point Likert scale, the respondents indicated a level of 4.08 (older adults: 4.33). A high number of respondents, including 78.1% (86.9% of older adults) declared either a high or a very high (4 or 5) level of safety perception in urban parks. In contrast, only 3.3% (1.4% of older adults) indicated either a very low or a low (1 or 2) level of safety. The Student’s *t*-test showed a statistically significant difference in the general perception of safety in public parks between these groups (*p* = 0.002, mean perceived security ratings for people under 60: 4.01, 60+: 4.33). The differences between the under 60 and 60+ aged respondents of the general perception of security in urban parks are presented in Figure 1.

The results of the mean perceived security ratings related to all safety-related factors taken into account in the survey are presented for all respondents, as well for all respondents divided into the under 60 and 60+ groups (see Table 3).

The first column in Table 3 presents the mean perceived security ratings for all respondents. Seven factors were indicated as very important (mean rate over 4.0). Three of them are connected to visibility (bright day, artificial lighting, and possibility to be visible), two are connected to external protection (police patrol and video surveillance), one was connected to maintenance and cleanliness (condition of equipment items), and one was connected to mobility facilities (park paths). Therefore, it can be indicated that in all discussed categories there are very important factors for the respondents’ sense of security. None of the factors achieved an average value lower than 2.5. One factor averaged responses between 2.5 and 3.0, and 11 of the 19 analyzed factors averaged between 3.0 and 4.0. The results show that the factors included in the survey were mostly considered by the respondents as important or very important.

The third and fourth columns of Table 3 present the differences in responses given by under 60 and 60+ aged park users. In general, the under 60 and 60+ aged respondents differently rated their overall safety level when visiting public parks, as well as the significance of most of the specific cases indicated in the questionnaire. When broken down by factor, it can be seen that older respondents generally gave more weight to factors that may have an impact on safety in all analyzed cases (all factors).

Out of all 19 factors, 9 were indicated by 60+ aged park users as particularly important for them (score 4.40 and higher) in the context of ensuring their safety in urban parks. From the mobility facilities category, the presence of park paths and functional aids (ramp, lift) was determined to be particularly important for the surveyed older adults. In the maintenance and cleanliness category, older adults indicated that the condition of pavement and equipment items were very important factors. In the visibility group of factors, the most important factors were identified to be the following: bright day and dark night, artificial lighting and possibility to be visible and to see others, i.e., those related to the presence or absence of light and the conditions for noticing potential threats. In the category of external protection, video surveillance was the only important factor; however, two other factors reached also high scores (over 4.30).

**Figure 2:**
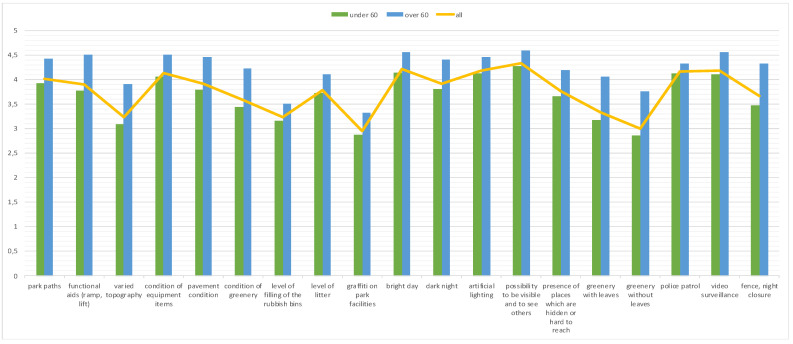
Summary of indications of the significance of factors influencing the perceived security of respondents by age (elaborated by authors).

The Student’s *t*-test was used to show the significance of differences in responses between under 60 and 60+ aged respondents (*p* < 0.05). As indicated in Table 3 (the fifth column), 18 of 19 indicated factors were statistically significant between these two groups. The biggest difference in the perception of factors affecting safety in urban parks was indicated in the case of pavement condition. Greenery with leaves was also much more important for older adults as compared to younger respondents. Significantly higher than the other categories of park users, people aged 60+ assessed the importance of factors such as functional aids (ramps, lifts) and varied topography that determine the safety and comfort of their physical movement in urban greenery. In the case of 8 factors, the *t*-value was over 4.0. Figure 2 shows a comparison of both groups of respondents (under 60 and 60+) with general results.

## 4. Discussion

The quality and attractiveness of urban parks, which translate directly to their use, depend on many aspects, with safety being one of the key factors, as confirmed by many studies [92,163,181]. According to Sundevall and Jansson [106], creating an inclusive public space appropriate for various age groups, including older adults, requires getting to know their habits and expectations. Therefore, shaping safe parks requires recognizing which factors are of the greatest importance for the users’ sense of security, especially those groups that are at risk of marginalization and exclusion, such as older adults. Contemporary shaping of urban green areas requires user-oriented design and management based upon a full understanding of the needs of all users [71,182,183]. Research on the perception of security by various groups of users is crucial for understanding their perspective and creating inclusive parks. This approach is currently being promoted [33,41,71,106,116,184,185,186,187] due to global aging trends. Conducting research on the safety of older adults in parks is also justified due to the need to create conditions for active aging [188].

The first hypothesis was that there are differences between the sense of security of older adults and other groups of urban park users. After literature review, we indicated that factors influencing the sense of security of all groups of users of urban green areas are discussed in publications to a very different extent [92,181]. In the case of reports regarding age, some of them show that older adults, included in the group of people with special requirements, generally have a lower sense of security than other age groups of users of public spaces [26,64,65,66,67,68,69]. However, the distinction in the perception of security between various age groups is not widely described.

The Student’s *t*-test used in this study to show the significance of the differences in responses between under 60 and 60+ aged respondents (*p* < 0.05) indicated that there was a difference in the case of 18 out of 19 factors. Only in the case of presence of police patrol in urban parks was there not statistically significant difference in perception among respondents under and over 60 years old. This proves that the needs of older adults differ from younger park users, which should have an impact on designing of urban parks.

The second hypothesis was that most factors related to aspects of the presence of mobility facilities, maintenance and cleanliness, visibility, and external protection are more important for the older adults’ sense of security, as they are a group of park users with special requirements. The confirmation of this hypothesis would allow the formulation of the statement that older adults pay more attention to security-providing factors than younger adults. The obtained results show that for all 19 factors, the importance assigned to them by older adults was greater than for the under 60 park users. The largest differences in the indications of the significance of individual factors between age groups (*t*-value above 4.0) occurred for eight factors: fence, pavement condition, greenery with and without leaves, functional aids, condition of greenery, varied topography, and condition of equipment. The results also allowed for the distinction of nine factors that have the greatest impact on the sense of security of older adults (score 4.40 and higher on the Likert scale). Among them were the presence of park paths and functional aids, the condition of pavement and equipment items, bright day and dark night, artificial lighting, the possibility to be visible and to see others, and video surveillance.

The obtained results are important because they firmly show the differences in the perception of safety between older and younger park users and at the same time prove that for older adults the factors influencing the sense of security are more important than for others. The conclusions from our study contribute to the development of research in this field, because there is very little cross-sectional research in the literature on the relationship between various factors occurring in parks and the sense of security of older adults.

In the mobility facilities category, selected factors (such as the presence of park paths and pavement condition) were identified by Wang and Rodiek [151] as particularly important for older adults. Other publications discussed the role of these elements, mainly in the context of ensuring the accessibility of urban parks, as well as the need to maintain them in good condition [26,116,122,123,151]. Only several studies have reported that not only the lack of paths, but also the uneven ground or poor-quality sidewalks are perceived as a problem for many park users [12,157]. However, there is not much current research on the direct link between the quality of path surfaces and their overall maintenance in parks in the context of the sense of security of older adults. It is also important to associate the perception of the sense of security resulting from this factor with another in this category, i.e., varied topography, which is discussed very rarely in the literature and mainly concerns the visual attractiveness of urban parks [160,161]. Only a few publications mention that general difficulties in moving around the park are one of the key factors affecting the sense of security [162]. The results of the research presented in this paper have also confirmed that the presence of varied topography is one of the factors important for the sense of security of older adults.

Functional aids (ramps, lifts) are also among the other factors that are particularly important for the sense of security of older adults, which was confirmed in our study. Their application is important for the proper functioning of public spaces in cities, ensuring their accessibility. However, most of the research in this area is in the context of designing spaces not related to green areas [23,189,190]. It also applies mainly to the disabled [191,192,193], and yet many able-bodied people lose their ease of movement and undergo height differences throughout their lives. Research in this area sporadically concerns green areas [26].

In the maintenance and cleanliness category, some research confirms that both of those aspects are generally important for adolescents and influence their visiting and physical activity in urban parks [45,60,187,188] and have an impact on older adults’ security [24,106,121,146]. The results related to the condition of park facilities, such as equipment and pavement, have been assessed as very important for the perception of security of 60+ aged users in our study. These findings are in line with the experiences presented by other studies [24,26,106,116,117,121,122,123,124]. The good condition of greenery, which makes the park visually attractive [57,80,108], is also crucial for older adults. This is mentioned in our study, as well as in other publications [24,92,106,179]. The results presented in publications show that all aspects of damage and litter usually have a significant impact on the sense of security of adolescent park users and older adults [30,97,106,135,136].

In the visibility category, factors such as bright day, dark night, and artificial lighting are generally recognized as having an impact on the sense of safety of all users, mainly in urban spaces, but less studied with regard to urban green areas [103]. However, in our research, two other factors from the visibility category (greenery with and without leaves) were among those showing the largest differences in the indications of significance of individual factors between age groups. At the same time, other research has not developed this field of study in the context of seniors.

The results related to the external protection category allowed to distinguish video surveillance as a particularly important factor for all respondents, not just older adults. This is consistent with the results of many studies conducted among various users of not only parks, but also public spaces in general [70,174,175,194,195,196,197,198]. At the same time in the opinion of older adults’, the use of cameras in urban green areas is perceived as an important strategy for maintaining a safe environment [45]; however, there is a lack of research on this relation. There is also no reference in any research to the importance of fencing parks and closing them at night, which was indicated in our study as important for the security perception of 60+ aged park users.

It is worth noting that although attention is drawn to the impact of various factors on the sense of security on users of public spaces [26,72,92,181], very little research is focused directly on older adults as users of urban parks, which is so important to improve both their health and physical condition. The results of our study indicate the importance of many factors for shaping both “age friendly” and “activity friendly” urban parks, which may provide safe and barrier-free green areas crucial to encouraging the physical activity of older adults’ [13]. They also confirm that the concerns of 60+ aged park users about their safety should be addressed with responsibility [26,45].

## 5. Conclusions

Creating urban green areas requires ensuring equal opportunities for different user groups and to avoiding potential conflicts between them. The global aging of urban populations is driving the adoption of age-friendly approaches. The challenge is to prepare for these changes in such a way that both current and future generations of older adults can fully benefit from these strategies. A special role in this respect is played by shaping urban parks, which are the basic places for recreation and improving health and well-being for older adults in cities. Urban parks that are not age diverse, among other factors, become uninviting and exclusive.

Safety is one of the most important aspects that influence the attractiveness of urban green areas and the tendency to use them for recreational purposes. This is confirmed by the research results presented in this study. They show that most of the factors discussed from all four categories (mobility facilities, maintenance and cleanliness, visibility, and external protection) are important for the safety of all user groups and are crucial for older adults due to the impact on their physical activity. This confirms hypothesis one. The results obtained in our study also confirm hypothesis two and show that 18 out of 19 factors related to aspects of the presence of mobility facilities, maintenance and cleanliness, visibility, and external protection are more important for older adults’ sense of security as a group of park users with special requirements.

The undertaken research is the stage on which to extend the knowledge supporting the design process of urban greenery that meet the expectations and needs of various user groups, in particular, those who are at risk of exclusion, such as older adults. When designing new parks and modernizing existing parks, it is important to make choices and implement solutions that, in responding to the needs of various user groups, both increase the attractiveness of green areas for the elderly and contribute even more to encouraging them to spend time outdoors, thus improving their health and well-being. For this to be possible, recommendations based on scientific research are needed.

The lack of detailed data confirms the legitimacy of undertaking our research. It also points out that it is important to identify in detail factors that determine safety in parks, both in younger people and older adults, and then to compare them with each other to reveal the differences. Insufficient knowledge on this subject, resulting in failure to meet the expectations of groups of people with special requirements, may, consequently, expose them to exclusion from the use of green areas, which are their primary place of rest, contact with nature, and improvement of health in highly urbanized areas. Therefore, it is important to continue research on older adults, increasing both the research sample and the number of factors which may affect their perception of security in urban parks.

The presented study makes a significant contribution to the completion of some knowledge gaps and helps to understand which factors characteristic to urban parks are important and affect the sense of security of older adults. This may, therefore, contribute to the improvement of the accessibility of urban parks, facilitate older adults’ engagement in physical activities, and thus enhance their health and well-being. This knowledge is needed, not only for researchers in theoretical terms, but above all it has a practical dimension and can be used as a tool supporting the process of planning and design of urban green areas to make them more inclusive, equitable, and to meet the expectations of all potential user groups, especially older adults.

## Figures and Tables

**Table 1:** Sample characteristics (elaborated by authors).

Variable	*n*	%	60+ Aged Park Users	% of 60+ Aged Park Users
**Age**				
18–29	124	31.31%		
30–44	101	25.51%		
45–59	100	25.25%		
60+	69	17.42%		
**Gender**				
male	137	34.8%	40	58.0%
female	257	65.2%	29	42.0%
**Place of living**				
village and small town	119	30.2%	26	37.7%
middle town and city	275	69.8%	43	62.3%
**Education**				
elementary	7	1.77%	1	1.4%
basic vocational education	13	3.28%	6	8.7%
secondary education	119	30.05%	31	44.9%
higher education	257	64.90%	31	44.9%
**Presence of urban parks in place of living**				
no parks or other green places	25	6.3%	1	1.4%
one	45	11.4%	6	8.7%
a few	324	82.2%	62	89.9%
**Frequency of use of urban parks**				
no use	6	1.5%	0	0%
couple of times per year	98	24.9%	14	20.3%
once a month	82	20.8%	19	27.5%
once per week	109	27.7%	25	36.2%
2–3 times per week	79	20.1%	9	13.0%
everyday	20	5.1%	2	2.9%

**Table 2:** Categories of factors indicated in questionnaire (elaborated by authors).

Category of Factors	Particular Factors
**Mobility facilities**	park paths functional aids (ramp, lift) varied topography
**Maintenance and cleanliness**	condition of equipment pavement condition condition of greenery level of filling the rubbish bins level of litter graffiti on park facilities
**Visibility**	bright day dark night artificial lighting possibility to be visible and to see others presence of hidden or hard to reach places greenery with leaves greenery without leaves
**External protection**	police patrol video surveillance fence, night closure

**Table 3:** Differences in responses between under 60 and 60+ aged park users (elaborated by authors).

Factor	Mean Perceived Security Ratings	Standard Deviation	Mean Perceived Security Ratings		*t*-Value	Significance
			under 60	60+		
**Mobility facilities**						
park paths	4.02	1.181	3.93	4.42	−3.665	*p* < 0.001
functional aids (ramp, lift)	3.90	1.268	3.77	4.51	−4.984	*p* < 0.001
varied topography	3.24	1.388	3.10	3.91	−4.832	*p* < 0.001
**Maintenance and cleanliness**						
condition of equipment items (benches and other rest equipment, litter bins, etc.)	4.13	1.091	4.06	4.51	−4.703	*p* < 0.001
pavement condition	3.91	1.174	3.79	4.46	−5.304	*p* < 0.001
condition of greenery	3.59	1.305	3.45	4.23	−4.957	*p* < 0.001
level of filling the rubbish bins	3.23	1.305	3.16	3.51	−2.590	*p* = 0.010
level of litter	3.79	1.189	3.72	4.10	−2.363	*p* = 0.018
graffiti on park facilities	2.95	1.394	2.87	3.33	−2.510	*p* = 0.012
**Visibility**						
bright day	4.21	1.213	4.14	4.55	−2.373	*p* = 0.018
dark night	3.91	1.345	3.80	4.40	−3.248	*p* = 0.001
artificial lighting	4.19	1.020	4.13	4.46	−2.628	*p* = 0.009
possibility to be visible and to see others	4.33	0.955	4.27	4.59	−2.691	*p* = 0.007
presence of hidden or hard to reach places	3.76	1.348	3.66	4.19	−3.215	*p* = 0.001
greenery with leaves	3.33	1.381	3.17	4.06	−5.148	*p* < 0.001
greenery without leaves	3.00	1.302	2.86	3.75	−4.994	*p* < 0.001
**External protection**						
police patrol	4.17	1.106	4.13	4.32	−0.949	*p* = 0.343
video surveillance	4.18	1.105	4.10	4.56	−3.549	*p* < 0.001
fence, night closure	3.67	1.351	3.47	4.33	−5.306	*p* < 0.001

## Data Availability

Data are contained within the article or available from referenced sources.

## References

[1] Peters K. (2010). Being together in Urban Parks: Connecting Public Space. Leis. Sci..

[2] Gill T.M., Allore H.G., Hardy S.E., Guo Z. (2006). The dynamic nature of mobility disability in older persons. J. Am. Geriatr. Soc..

[3] (2016). World Health Assembly, 69. Multisectoral Action for a Life Course Approach to Healthy Ageing: Draft Global Strategy and Plan of Action on Ageing and Health: Report by the Secretariat.

[4] Wyszkowska D., Gabińska M., Romańska S. (2021). Sytuacja Osób Starszych w Polsce w 2019 r (The Situation of Older People in Poland in 2019).

[5] Plouffe L., Kalache A. (2010). Towards global age-friendly cities: Determining urban features that promote active aging. J. Urban Health.

[6] Van Hoof J., Kazak J.K., Perek-Białas J.M., Peek S. (2018). The challenges of urban ageing: Making cities age-friendly in Europe. Int. J. Environ. Res. Public Health.

[7] (2007). Global Age-Friendly Cities: A Guide.

[8] Gill T.M., Gahbauer E.A., Murphy T.E., Han L., Allore H.G. (2012). Risk Factors and Precipitants of Long-Term Disability in Community Mobility: A Cohort Study of Older Persons. Ann. Intern. Med..

[9] Manini T.M. (2013). Mobility decline in old age: A time to intervene. Exerc. Sport Sci. Rev..

[10] Rantanen T. (2013). Promoting mobility in older people. J. Prev. Med. Public Health..

[11] Miyashita T., Kudo S., Maekawa Y. (2021). Assessment of walking disorder in community-dwelling Japanese middle-aged and elderly women using an inertial sensor. PeerJ.

[12] Ottoni C.A., Sims-Gould J., Winters M. (2021). Safety perceptions of older adults on an urban greenway: Interplay of the social and built environment. Health Place.

[13] Zhai Y., Li D., Wang D., Shi C. (2020). Seniors’ Physical Activity in Neighborhood Parks and Park Design Characteristics. Front. Public Health.

[14] Bauman A.E., Reis R.S., Sallis J.F., Wells J.C., Loos R.J.F., Martin B.W. (2012). Correlates of physical activity: Why are some people physically active and others not?. Lancet.

[15] Thompson C.W. (2002). Urban open space in the 21st century. Landsc. Urban Plan..

[16] Payne L., Orsega-Smith E., Roy M., Godbey G. (2005). Local park use and personal health among older adults: An exploratory study. J. Park Recreat. Admin..

[17] Cohen D.A., McKenzie T.L., Sehgal A., Williamson S., Golinelli D., Lurie N. (2007). Contribution of public parks to physical activity. Am. J. Public Health.

[18] Sugijama T., Ward Thompson C. (2008). Associations between characteristics of neighbourhood open space and older people’s walking. Urban For. Urban Green..

[19] Rosenberger R.S., Bergerson T.R., Kline J.D. (2009). Macro-Linkages between Health and Outdoor Recreation: The Role of Parks and Recreation Providers. J. Parks Recreat. Admin..

[20] Santos T., Nogueira Mendes R., Vasco A. (2016). Recreational activities in urban parks: Spatial interactions among users. J. Outdoor Recreat. Tour..

[21] Kaczynski A., Henderson K.A. (2007). Environmental correlates of physical activity: A review of evidence about parks and recreation. Leis. Sci..

[22] Kaplan R., Kaplan S. (1989). The Experience of Nature: A Psychological Perspective.

[23] Yung E.H.K., Conejos S., Chan E.H.W. (2016). Social needs of the elderly and active aging in public open spaces in urban renewal. Cities.

[24] Wen C., Albert C., Von Haaren C. (2018). The elderly in green spaces: Exploring requirements and preferences concerning nature-based recreation. Sustain. Cities Soc..

[25] Gikwad A., Shinde K.A. (2019). Use of parks by older persons and perceived health benefits: A developing country context. Cities.

[26] Onose D.A., Ioja I.C., Nita M.R., Vânau G.O., Popa A.M. (2020). Too Old for Recreation? How Friendly Are Urban Parks for Elderly People?. Sustainability.

[27] Ulrich R.S., Addoms D.L. (1981). Psychological and Recreational Benefits of a Residential Park. J. Leis. Res..

[28] Kweon B.S., Sullivan W.C., Wiley A.R. (1998). Green Common Spaces and the Social Integration of Inner-City Older Adults. Environ. Behav..

[29] Humpel N., Owen N., Leslie E. (2002). Environmental factors associated with adults’ participation in physical activity: A review. Am. J. Prev. Med..

[30] Bedimo-Rung A.L., Mowen A.J., Cohen D.A. (2005). The significance of parks to physical activity and public health: A conceptual model. Am. J. Prev. Med..

[31] Kemperman A., Timmermans H. (2014). Green spaces in the direct living environment and social contacts of the aging population. Landsc. Urban Plan..

[32] Barnett D.W., Barnett A., Nathan A., van Cauwenberg J., Cerin E. (2017). Built environmental correlates of older adults’ total physical activity and walking: A systematic review and meta-analysis. Int. J. Behav. Nutr. Phys. Act..

[33] Yung E.H.K., Ho W.K.O., Chan E.H.W. (2017). Elderly satisfaction with planning and design of public parks in high density old districts: An ordered logit model. Landsc. Urban Plan..

[34] Enssle F., Kabisch N. (2020). Urban green spaces for the social interaction, health and well-being of older—An integrated view of urban ecosystem services and socio-environmental justice. Environ. Sci. Policy.

[35] Maller C., Townsend M. (2002). Healthy Parks Healthy People: The Health Benefits of Contact with Nature in a Park Context: A Review of Current Literature. Social and Mental Health Priority Area Occasional Paper Series, 1; Parks Victoria, Deakin University Faculty of Health & Behavioral Sciences. http://hdl.handle.net/10536/DRO/DU:30010146.

[36] Chiesura A. (2004). The Role of Urban Parks for the Sustainable City. Landsc. Urban Plan..

[37] Rappe E., Kivela S.L., Rita H. (2006). Visiting outdoor green environments positively impacts self-rated health among older people in long-term care. Horttechnology.

[38] Tzoulas K., Korpela K., Venn S., Yli-Pelkonen V., Kaz’miercak A., Niemela J., James P. (2007). Promoting ecosystem and human health in urban areas using greens pace infrastructure: A literature review. Landsc. Urban Plan..

[39] Chodzko-Zajko W., Schwingel A., Chae Hee P. (2008). Successful aging: The role of physical activity. Am. J. Lifestyle Med..

[40] Konijnendijk C.C., Annerstedt M., Nielson A.B., Maruthaveeran S. (2013). Benefits of Urban Parks: A Systematic Review.

[41] Wolch J.R., Byrne J., Newell J.P. (2014). Urban green space, public health, and environmental justice: The challenge of making cities ‘just green enough’. Landsc. Urban Plan..

[42] Kuo M. (2015). How might contact with nature promote human health? Promising mechanisms and a possible central pathway. Front. Psychol..

[43] Cunningham C., O’Sullivan R., Caserotti P., Tully M.A. (2020). Consequences of physical inactivity in older adults: A systematic review of reviews and meta-analyses. Scand. J. Med. Sci. Sports.

[44] Loukaitou-Sideris A., Mehta V., Palazzo D. (2020). Designing parks for older adults. Companion to Public Space.

[45] Mertens L., Van Cauwenberg J., Veitch J., Deforche B., Van Dyck D. (2019). Differences in park characteristic preferences for visitation and physical activity among adolescents: A latent class analysis. PLoS ONE.

[46] Maslow A. (1990). Motywacja i Osobowość.

[47] Bar-Tal D. (2000). Shared Beliefs in a Society: Social Psychological Analysis.

[48] Bańka A. (2002). Społeczna Psychologia Środowiskowa.

[49] Braithwaite V. (2009). Security and Harmony Value Orientations and Their Roles in Attitude Formation and Change. Psychol. Inq..

[50] Pomykała M. (2010). Bezpieczeństwo—W poszukiwaniu definicji. Zesz. Nauk. Politech. Rzesz. Zarządzanie I Mark..

[51] Schwartz S.H. (2012). An Overview of the Schwartz Theory of Basic Values. Online Read. Psychol. Cult..

[52] Bar-Tal D., Jacobson D. (1998). A Psychological Perspective on Security. Appl. Psychol. An. Int. Rev..

[53] Schwartz S.H. (2002). Value Priorities and Behavior: Applying a Theory of Integrated Value Systems. Psicodebate.

[54] Nęcka E., Orzechowski J., Szymura B. (2020). Psychologia Poznawcza.

[55] Maas J., Spreeuwenberg P., van Winsum-Westra M., Verheij R.A., Vries S., Groenewegen P.P. (2009). Is green space in the living environment associated with people’s feelings of social safety?. Environ. Plan..

[56] Mehta V. (2014). Evaluating public space. J. Urban Des..

[57] Kuo F.E., Bacaicoa M., Sullivan W.C. (1998). Transforming inner-city landscapes. Environ. Behav..

[58] Ode Sang Å., Knez I., Gunnarsson B., Hedblom M. (2016). The effects of naturalness, gender, and age on how urban green space is perceived and used. Urban For. Urban Green..

[59] Laatikainen T.E., Broberg A., Kyttä M. (2017). The physical environment of positive places: Exploring differences between age groups. Prev. Med..

[60] Veitch J., Salmon J., Deforche B., Ghekiere A., Van Cauwenberg J., Bangay S., Timperio A. (2017). Park attributes that encourage park visitation among adolescents: A conjoint analysis. Landsc. Urban Plan..

[61] Hong A., Sallis J.F., King A.C., Conway T.L., Saelens B., Cain K.I., Fox E.H., Frank L.D. (2018). Linking green space to neighborhood social capital in older adults: The role of perceived safety. Soc. Sci. Med..

[62] Jansson M., Fors H., Lindgren T., Wiström B. (2013). Perceived personal safety in relation to urban woodland vegetation—A review. Urban For. Urban Green..

[63] Wiles J.L., Leibing A., Guberman N., Reeve J., Allen R.E. (2012). The meaning of “aging in place” to older people. Gerontologist.

[64] Burgess J. (1996). Focusing on fear: The use of focus groups in a project for the Community Forest Unit, Countryside Commission. Area.

[65] Madge C. (1997). Public parks and the geography of fear. Tijdschrift voor Economische en Sociale Geografie.

[66] Lynch G., Atkins S. (1988). The influence of personal security fears on women’s travel patterns. Transportation.

[67] James K., Embrey L. (2001). ‘Anyone could be lurking around!’: Constraints on adolescent girls’ recreational activities after dark. World Leis. J..

[68] Diprose R. (2007). Physical Safety and Security: A Proposal for Internationally Comparable Indicators of Violence. Oxf. Dev. Stud..

[69] Murray S.L., Holmes J.G., Griffin D.W. (2000). Self-esteem and the quest for felt security: How perceived regard regulates attachment processes. J. Personal. Soc. Psychol..

[70] Lindquist J.H., Duke J.M. (1982). The elderly victim at risk: Explaining the fear-victimization paradox. Criminology.

[71] Jansson N., Fors H., Sundevall E.P., Bengtsson A., Lerstrup I., Hurley P., Qviström M., Randrup T.B., Jansson M., Randrup T. (2020). User-oriented urban open space governance and management. Urban Open Space Governance and Management.

[72] Mahrous A.M., Mustafa Y.M., Abou El-Ela M.A. (2018). Physical characteristics and perceived security in urban parks: Investigation in the Egyptian context. Ain Shams Eng. J..

[73] Loewen J.L., Steel G.D., Suedfeld P. (1993). Perceived safety from crime in the urban environment. J. Environ. Psychol..

[74] Crew K. (2001). Linear Parks and Urban Neighborhoods: A Study of the Crime Impact of the Boston South-west Corridor. J. Urban Des..

[75] Welsh B.P., Farrington D.C. (2008). Effects of improved street lighting on crime. Campbell Syst. Rev..

[76] Lorenc T., Petticrew M., Whitehead M., Neary D., Clayton S., Wright K., Renton A. (2013). Environmental interventions to reduce fear of crime: Systematic review of effectiveness. Syst. Rev..

[77] Fotios S.A., Unwin J., Farrall S. (2015). Road lighting and pedestrian reassurance after dark: A review. Light. Res. Technol..

[78] Lab S.P. (2019). Crime Prevention: Approaches, Practices and Evaluations.

[79] Appleton J. (1975). The Experience of Landscape.

[80] Schroeder H., Anderson L. (1984). Perception of personal safety in urban recreation sites. J. Leis. Res..

[81] Nasar J.L., Fisher B.S. (1993). “Hot spots” of fear and crime: A multi-method investigation. J. Environ. Psychol..

[82] Herzog T., Kutzli H. (2002). Preference and perceived danger in field/forest settings. Environ. Behav..

[83] Bennett G.G., McNeill L.H., Wolin K.Y., Duncan D.T., Puleo E., Emmons K.M. (2007). Safe To Walk? Neighborhood Safety and Physical Activity Among Public Housing Residents. PLoS Med..

[84] Fox K.A., Nobles M.R., Piquero A.R. (2009). Gender, crime victimization and fear of crime. Secur. J..

[85] van Rijswijk L., Haans A. (2018). Illuminating for safety: Investigating the role of lighting Appraisals on the Perception of Safety in the Urban Environment. Environ. Behav..

[86] Rahm J., Sternudd C., Johansson M. (2021). In the evening, I don’t walk in the park: The interplay between street lighting and greenery in perceived safety. Urban Des. Int..

[87] Nasar J., Jones K. (1997). Landscape of fear and stress. Environ. Behav..

[88] Fisher B., May D. (2009). College students’ crime-related fear on campus: Are fear provoking cues gendered?. J. Contemp. Crim. Justice.

[89] Yeoh B.S.A., Yeow P.L. (1997). Where women fear to tread: Images of danger and the effects of fear of crime in Singapore. GeoJournal.

[90] Brownlow A. (2006). An archaeology of fear and environmental change in Philadelphia. Geoforum.

[91] Boomsma C., Steg L. (2014). Feeling safe in the dark: Examining the effect of entrapment, lighting levels, and gender on feelings of safety and lighting policy acceptability. Environ. Behav..

[92] Maruthaveeran S., Konijnendijk van den Bosch C.C. (2014). A socio-ecological exploration of fear of crime in Urban green spaces—A systematic review. Urban For. Urban Green..

[93] Fineschi S., Loreto F. (2020). A Survey of Multiple Interactions Between Plants and the Urban Environment. Front. For. Glob. Change.

[94] Painter K. (1996). The influence of street lighting improvements on crime, fear and pedestrian street use after dark. Landsc. Urban Plann..

[95] Shenassa E., Liebhaber A., Ezeamama A. (2006). Perceived safety of area of residence and exercise: A pan-European study. Am. J. Epidemiol..

[96] Mani M., Abdullah A., Azam M., Jayaraman K., Bagheri A. (2012). The importance of well-designed children’s play-environments in reducing parental concerns. Middle-East J. Sci. Res..

[97] Maruthaveeran S., Konijnendijk van den Bosch C.C. (2015). Fear of crime in urban parks—What the residents of Kuala Lumpur have to say?. Urban For. Urban Green..

[98] Orr N., Wagstaffe A., Briscoe S., Garside R. (2016). How do older people describe their sensory experiences of the natural world? A systematic review of the qualitative evidence. BMC Geriatr..

[99] Schroeder H.W., Zube E.H., Moore G.T. (1989). Environment, behavior and design research on urban forests. Advances in Environment, Behavior and Design.

[100] Andrews M., Gatersleben B. (2010). Variations in perception of danger, fear and preference in a simulated natural environment. J. Environ. Psychol..

[101] Lis A., Pardela Ł., Iwankowski P. (2019). Impact of Vegetation on Perceived Safety and Preference in City Parks. Sustainability.

[102] Gao T., Liang H., Chen Y., Qiu L. (2019). Comparisons of Landscape Preferences through Three Different Perceptual Approaches. Int. J. Environ. Res. Public Health.

[103] Kimic K., Polko P., Fialová J. (2021). Perception of natural elements by park users in the context of security. Proceedings of the Public Recreation and Landscape Protection—With Sense Hand in Hand!.

[104] Burgess J., Harrison C.M., Limb M. (1988). People, parks and the urban green: A study of popular meanings and values for open spaces in the city. Urban Stud..

[105] Knutsson J., Homel R. (1997). Restoring public order in a city Park. Policing for Prevention: Reducing Crime, Public Intoxication and Injury.

[106] Sundevall E.P., Jansson M. (2020). Inclusive Parks across Ages: Multifunction and Urban Open Space Management for Children, Adolescents, and the Elderly. Int. J. Environ. Res. Public Health.

[107] Talbot J., Kaplan R. (1984). Needs and fears: The response to trees and nature in the inner city. J. Arboric..

[108] Jorgensen A., Hitchmough J., Calvert T. (2002). Woodland spaces and edges: Their impact on perception of safety and preference. Landsc. Urban Plan..

[109] Wolfe M., Mennis J. (2012). Does vegetation encourage or suppress urban crime? Evidence from Philadelphia, PA. Landsc. Urban Plan..

[110] Qiu L., Lindberg S., Nielsen A.B. (2013). Is biodiversity attractive? On-site perception of recreational and biodiversity values in urban green space. Landsc. Urban Plan..

[111] Jorgensen A., Anthopoulou A. (2007). Enjoyment and fear in urban woodlands—Does age make a difference?. Urban For. Urban Green..

[112] Li F., Fisher J., Brownson R.C., Bosworth M. (2005). Multilevel modeling of built environment characteristics related to neighbourhood walking activity in older adults. J. Epidemiol. Community Health.

[113] Pfisterir M. (2002). Understanding Crime and Perceptions of Safety in Providence’s Parks. Undergraduate Thesis.

[114] Alves S., Aspinall P.A., Ward Thompson C., Sugiyama T., Brice R., Vickers A. (2008). Preferences of older people for environmental attributes of local parks: The use of choice-based conjoint analysis. Facilities.

[115] Hilborn J. (2009). Dealing with Crime and Disorder in Urban Parks.

[116] Wu K.C., Song L.Y. (2017). A case for inclusive design: Analyzing the needs of those who frequent Taiwan’s urban parks. Appl. Ergon..

[117] Huang T., Huang C. (2019). Study on the preference of senior citizens in urban park public facilities. Int. J. Soc. Sci..

[118] Shaffer G.S., Anderson L.M. (1985). Perceptions of the Security and Attractiveness of Urban Parking Lots. J. Environ. Psychol..

[119] Gearin E., Kahle C. (2006). Teen and adult perceptions of urban green space Los Angeles. Child. Youth Environ..

[120] Abdelhamid M.M., Elfakharany M.M. (2020). Improving urban park usability in developing countries: Case study of Al-Shalalat Park in Alexandra. Alex. Eng. J..

[121] Lindberg M., Schipperijn J. (2015). Active use of urban park facilities—Expectations versus reality. Urban For. Urban Green..

[122] Zhai Y., Baran P.K. (2016). Do configurational attributes matter in context of urban parks? Park pathway configurational attributes and senior walking. Landsc. Urban Plan..

[123] Zhai Y., Baran P.K. (2017). Urban park pathway design characteristics and senior walking behavior. Urban For. Urban Green..

[124] Błaszczyk M., Suchocka M., Gawłowska A., Kimic K., Kaszuba K., Fialová J. (2019). Warsaw parks as recreational places: Needs and preferences of the elderly users. Proceedings of the Public Recreation and Landscape Protection—With Sense Hand in Hand!.

[125] Levinger P., Panisset M., Parker H., Batchelor F., Tye M., Hill K.D. (2021). Guidance about age-friendly outdoor exercise equipment and associated strategies to maximise usability for older people. Health Promot. J. Aust. Off. J. Aust. Assoc. Health Promot. Prof..

[126] Corazza M.V., Di Mascio P., Moretti L. (2018). Management of sidewalk maintenance to improve walking comfort for senior citizens. WIT Trans. Built Environ..

[127] Chow H. (2013). Outdoor fitness equipment in parks: A qualitative study from older adults’ perceptions. BMC Public Health.

[128] Copeland J.L., Currie C., Walker A., Mason E., Willoughby T.N., Amson A. (2017). Fitness Equipment in Public Parks: Frequency of Use and Community Perceptions in a Small Urban Centre. J. Phys. Act. Health..

[129] Bixler R.D., Floyd M.F. (1997). Nature is Scary, Disgusting, and Uncomfortable. Environ. Behav..

[130] Wilson E.O. (1984). Biophilia.

[131] Braverman I. (2008). Everybody loves trees: Policing American Cities Through Street Trees. Duke Environ. Law Policy Forum.

[132] Mullaney J., Lucke T., Trueman S.J. (2015). A review of benefits and challenges in growing street trees in paved Urban environments. Landsc. Urban Plan..

[133] Suchocka M., Kimic K., Fialová J. (2019). Management of urban forest to ensure the safety of touristic use on the example of Warsaw. Proceedings of the Public Recreation and Landscape Protection—With Sense Hand In hand!.

[134] Thani S.K.S.O., Hashim N., Ismail W. (2016). Surveillance by Design: Assessment Using Principles of Crime Prevention through Environmental Design (CPTED) in Urban Parks. Procedia Soc. Behav. Sci..

[135] LaGrange R.L., Ferraro K.F., Supancic M. (1992). Perceived risk and fear of crime: Role of social and physical incivilities. J. Res. Crime Delinquency.

[136] Stefanizzi S., Verdolini V. (2019). Bordered communities: The perception of insecurity in five European cities. Qual. Quant. Int. J. Methol..

[137] Corti B., Donovan R., Holman C. (1996). Factors influencing the use of physical activity facilities: Results from qualitative research. Health Promot. J. Aust..

[138] Bai H., Wilhelm Stanis S.A., Kaczynski A.T., Besenyi G.M. (2013). Perceptions of neighborhood park quality: Associations with physical activity and body mass index. Ann. Behav. Med..

[139] Zhang R., Wulff H., Duan Y., Wagner P. (2019). Associations between the physical environment and park-based physical activity: A systematic review. J. Sport Health Sci..

[140] Wilson J.Q., Kelling G.L., Cullen F.T., Wilcox P. (2010). Broken Windows Theory. Encyclopedia of Criminological Theory.

[141] Bahriny F., Bell S. (2020). Patterns of Urban Park Use and Their Relationship to Factors of Quality: A Case Study of Tehran, Iran. Sustainability.

[142] What Role Can Maintenance and Operations Play in Creating Safer Parks? Project for Public Spaces. https://www.pps.org/article/torontosafety4.

[143] Robinson J.B., Lawton B.A., Taylor R.B., Perkins D.D. (2003). Multilevel longitudinal impacts of incivilities: Fear of crime, expected safety, and block satisfaction. J. Quant. Criminol..

[144] McCormack G.R., Rock M., Toohey A.M., Hignell D. (2010). Characteristics of urban parks associated with park use and physical activity: A review of qualitative research. Health Place.

[145] Bertram C., Rehdanz K. (2015). Preferences for cultural urban ecosystem services: Comparing attitudes, perception, and use. Ecosyst. Serv..

[146] Arnberger A., Eder R. (2011). The influence of age on recreational trail preferences of urban green-space visitors: A discrete choice experiment with digitally calibrated images. J. Environ. Plan. Manag..

[147] Kaczynski A.T., Potwarka L.R., Saelens B.E. (2008). Association of park size, distance, and features with physical activity in neighborhood parks. Am. J. Public Health.

[148] Aspinall P.A., Thompson C., Alves W., Sugiyama S., Brice T.R., Vickers A. (2010). Preference and relative importance for environmental attributes of neighborhood open space in older people. Environ. Plan. B Plan. Des..

[149] Choi Y.J., Matz-Costa C. (2017). Perceived neighborhood safety, social cohesion, and psychological health of older adults. Gerontologist.

[150] Haber D. (2016). Health Promotion and Aging: Practical Applications for Health Professionals.

[151] Wang X., Rodiek S. (2019). Older Adults’ Preference for Landscape Features Along Urban Park Walkways in Nanjing, China. Int. J. Environ. Res. Public Health.

[152] Veitch J., Flowers E., Ball K., Deforche B., Timperio A. (2020). Designing parks for older adults: A qualitative study using walk-along interviews. Urban For. Urban Green..

[153] Sallis J.F., Cervero R.B., Ascher W., Henderson K.A., Kraft M.K., Kerr J. (2006). An ecological approach to creating active living communities. Annu. Rev. Public Health.

[154] van Dyck D., Teychenne M., McNaughton S.A., Bourdeaudhuij I., de Salmon J., Huerta-Quintanilla R. (2015). Relationship of the perceived social and physical environment with mental health-related quality of life in middle-aged and older adults: Mediating effects of physical activity. PLoS ONE.

[155] Hirvensalo M., Rantanen T., Heikkinen E. (2000). Mobility difficulties and physical activity as predictors of mortality and loss of independence in the community-living older population. J. Am. Geriatr. Soc..

[156] Delbaere K., Crombez G., Vanderstraeten G., Willems T., Cambier D. (2004). Fear-related avoidance of activities, falls and physical frailty. A prospective community-based cohort study. Age Ageing.

[157] Clarke P., George L.K. (2005). The role of the built environment in the Disablement Process. Am. J. Public Health.

[158] Hung K., Crompton J.L. (2006). Benefits and Constraints Associated with the Use of an Urban Park Reported by a Sample of Elderly in Hong Kong. Leis. Stud..

[159] Kempen G.I., van Haastregt J.C., McKee K.J., Delbaere K., Zijlstra G.R. (2009). Socio-demographic, health-related and psychosocial correlates of fear of falling and avoidance of activity in community-living older persons who avoid activity due to fear of falling. BMC Public Health.

[160] Rasidi M.H., Jamirsah N., Said I. (2012). Urban Green Space Design Affects Urban Residents’ Social Interaction. Procedia Soc. Behav. Sci..

[161] Van Vliet E., Dane G., Weijs-Perrée M., van Leeuwen E., van Dinter M., van den Berg P., Borgers A., Chamilothori K. (2021). The Influence of Urban Park Attributes on User Preferences: Evaluation of Virtual Parks in an Online Stated-Choice Experiment. Int. J. Environ. Res. Public Health.

[162] Martin Ginis K.A., Ma J.K., Latimer-Cheung A.E., Rimmer J.H. (2016). A systematic review of review articles addressing factors related to physical activity participation among children and adults with physical disabilities. Health Psychol. Rev..

[163] Park K. (2017). Psychological park accessibility: A systematic literature review of perceptual components affecting park use. Landsc. Res..

[164] Moulaert T., Wanka A. (2019). Benches as Materialisations of (Active) Ageing in Public Space: First Steps towards a Praxeology of Space. Urban Plan..

[165] Turel H.S., Yigit E.M., Altug I. (2007). Evaluation of elderly people’s requirements in public open spaces: A case study in Bornova District (Izmir, Turkey). Build. Environ..

[166] Perry M., Cotes L., Horton B., Kunac R., Snell I., Taylor B., Wright A., Devan H. (2021). “Enticing” but Not Necessarily a “Space Designed for Me”: Experiences of Urban Park Use by Older Adults with Disability. Int. J. Environ. Res. Public Health.

[167] Lis A., Pardela Ł., Can W., Katlapa A., Rąbalski Ł. (2019). Perceived Danger and Landscape Preferences of Walking Paths with Trees and Shrubs by Women. Sustainability.

[168] Stamps A.E. (2005). Enclosure and Safety in Urbanscapes. Environ. Behav..

[169] Shi S., Gou Z., Chen L.H.C. (2014). How does enclosure influence environmental preferences? A cognitive study on urban public open spaces in Hong Kong. Sustain. Cities Soc..

[170] Nordh H., Østby K. (2013). Pocket parks for people—A study of park design and use. Urban For. Urban Green..

[171] Welsh B.C., Farrington D.P. (2009). Public area CCTV and crime prevention: An updated systematic review and meta-analysis. Justice Q..

[172] Lee J.S., Park S., Jung S. (2016). Effect of Crime Prevention through Environmental Design (CPTED) Measures on Active Living and Fear of Crime. Sustainability.

[173] Atlas R.I. (2013). 21st Century Security and CPTED: Designing for Critical Infrastructure, Protection and Crime Prevention.

[174] Socha R., Kogut B. (2020). Urban Video Surveillance as a Tool to Improve Security in Public Spaces. Sustainability.

[175] Cozens P.M., Saville G., Hillier D. (2005). Crime prevention through environmental design (CPTED): A review and modern bibliography. Prop. Manag..

[176] McCormick J., Holland S. (2015). Strategies in use to reduce incivilities, provide security and reduce crime in urban parks. Secur. J..

[177] Iqbal A., Ceccato V. (2016). Is CPTED useful to guide the inventory of safety in parks? A study case in Stockholm, Sweden. Int. Crim. Justice Rev..

[178] Bogacka E., Ceccato V., Nalla M.K. (2020). Safety of urban park users. The case of Poznań, Poland. Crime and Fear in Public Places. Towards Safe, Inclusive and Sustainable Cities.

[179] Polko P., Kimic K. (2022). Gender as a factor differentiating the perceptions of safety in urban parks. Ain Shams Eng. J..

[180] Likert R.A. (1932). Technique for the Measurement of Attitudes. Arch. Psychol..

[181] Mak B.K.L., Jim C.Y. (2021). Contributions of human and environmental factors to concerns of personal safety and crime in urban parks. Secur. J..

[182] Mak B.K.L., Jim C.Y. (2018). Examining fear-evoking factors in urban parks in Hong Kong. Landsc. Urban Plan..

[183] Haaland C., van den Bosch C.K. (2015). Challenges and strategies for urban green-space planning in cities undergoing densification: A review. Urban For. Urban Green..

[184] Gholipour S., MahdiNejad J.e.D., Saleh Sedghpour B. (2021). Security and urban satisfaction: Developing a model based on safe urban park design components extracted from users’ preferences. Secur. J..

[185] Mehta V., Mahato B. (2020). Designing urban parks for inclusion, equity, and diversity. J. Urban Int. Res. Placemaking Urban Sustain..

[186] Landman K. (2020). Inclusive public space: Rethinking practices of mitigation, adaptation and transformation. Urban Des. Int..

[187] Iqbal A. (2021). Inclusive, Safe and Resilient Public Spaces: Gateway to Sustainable Cities?.

[188] Van Hecke L., Deforche B., van Dyck D., De Bourdeaudhuij I., Veitch J., Van Cauwenberg J. (2016). Social and physical environmental factors influencing adolescents’ physical activity in urban public open spaces: A qualitative study using walk-along interviews. PLoS ONE.

[189] Veitch J., Salmon J., Parker K., Bangay S., Deforche B., Timperio A. (2016). Adolescents’ ratings of features of parks that encourage park visitation and physical activity. Int. J. Behav. Nutr. Phys. Act..

[190] Loukaitou-Sideris A., Levy-Storms L., Brozen M. (2014). Placemaking for an Ageing Population: Guidelines for Senior-Friendly Parks.

[191] Loukaitou-Sideris A., Levy-Storms L., Chen L., Brozen M. (2016). Parks for an Aging Population: Needs and Preferences of Low-Income Seniors in Los Angeles. J. Am. Plan. Assoc..

[192] Hasanvand S., Ebrahimpour M., Bagheri B., Razmkhah S., Khojasteh Ghamari M.A. (2014). Improving of urban public spaces safety in order to using physical disabled persons. Int. J. Civil. Eng. Constr. Estate Manag..

[193] Sertaç G., Metin D., Tuğrul P.A. (2016). A Research on accessibility of Urban Parks by disabled users. Int. J. Res. Soc. Sci..

[194] Ratcliffe J.H., Taniguchi T., Taylor R.B. (2009). The Crime Reduction Effects of Public CCTV Cameras: A Multi-Method Spatial Approach. Justice Q..

[195] Wieteska-Rosiak B. (2015). Kształtowanie przestrzeni publicznej z uwzględnieniem aspektów bezpieczeństwa publicznego. Stud. KPZK PAN.

[196] Reaves B.A. (2015). Local Police Departments. Equipment and Technology.

[197] Gerell M. (2016). Hot Spot Policing With Actively Monitored CCTV Cameras: Does it Reduce Assaults in Public Places?. Int. Crim. Justice Rev..

[198] Piza E.L., Welsh B.C., Farrington D.P., Thomas A.L. (2019). CCTV Surveillance for crime prevention: A 40-year systematic review with meta-analysis. Criminol. Public Policy.

